# Effects of Air Pollution on the Health of Older Adults during Physical Activities: Mapping Review

**DOI:** 10.3390/ijerph20043506

**Published:** 2023-02-16

**Authors:** Alexandro Andrade, Anderson D’Oliveira, Loiane Cristina De Souza, Ana Cecilia Rosatelli de Freitas Bastos, Fábio Hech Dominski, Luca Stabile, Giorgio Buonanno

**Affiliations:** 1 Health and Sports Science Center, Department of Physical Education, CEFID, Santa Catarina State University, Florianópolis 88035-901, Brazil; 2 Department of Physical Education, Univille University, Joinville 89219-710, Brazil; 3 Department of Civil and Mechanical Engineering, University of Cassino and Southern Lazio, Via Di Biasio 43, 03043 Cassino, Italy; 4 International Laboratory for Air Quality and Health, Queensland University of Technology, Brisbane 4001, Australia

**Keywords:** aging, exercise, evidence-based review, environment and public health

## Abstract

Atmospheric pollutants present environmental threats to health and have been investigated in different environments, such as highways, squares, parks, and gyms. These environments are frequented by older adults, who are considered fragile to the harmful impacts of pollution present in the air. The aim was to analyze the state of the art on the effects of air pollution on the health of older adults during physical activities (PAs) through a mapping review. The search was performed in PubMed, Web of Science, Scopus, and Cinahl databases until June 2022. Of the 10,109 studies initially identified, 58 met the inclusion criteria. The most investigated health outcome was cardiovascular disease, followed by respiratory outcomes. Particulate matter (PM_2.5_ and PM_10_), nitrogen dioxide (NO_2_), and ozone (O_3_) were the most investigated pollutants. Of the 75 health outcomes investigated, in 29, air pollution had harmful effects on the health of the older adults during the practice of PA, more frequently in cardiovascular diseases. In 25 outcomes, the beneficial effects of PA to the health of the older adults remained, despite exposure to high and low concentrations of pollutants, most often in terms of mental disorders. We conclude that poor air quality is a harmful factor for the health of older adults during the practice of PAs, more frequently in cardiovascular and respiratory diseases. On the other hand, for mental-health-related outcomes (depression and cognition), in most studies, the beneficial effects of PA in older adults were maintained, even after exposure to pollutants.

## 1. Introduction

Air pollution is one of the greatest environmental threats to human health. The main pollutants that present health risks are particulate matter (PM), ozone (O₃), nitrogen dioxide (NO₂), sulfur dioxide (SO₂), and carbon monoxide (CO) [1], which have been investigated in different environments, such as residential kitchens, highways, squares, parks, and gyms [1,2]. These environments are frequented by different populations, such as older adults. Today, this population is considered the most fragile to the harmful impacts of pollution present in the air [1] due to the entry of these pollutants deep into the lungs and bloodstreams, traveling through the organs of the human body, causing severe tissue and cell damage [1].

Global assessments suggest that air pollution is an all-cause mortality risk [3]. Among people aged 60 years and over, the greatest impacts on health and mortality levels are observed in individuals who already have chronic diseases such as asthma, chronic obstructive pulmonary disease (COPD), and heart disease [3]. Healthy practices can contribute to the reduction in reported diseases and mortality levels, such as physical activities (PAs); however, there is evidence that air pollution affects PA behavior, canceling out some or all of the benefits of the practice [4].

The literature contains many studies demonstrating the favorable health effects of physical exercise, with it being recommended for different age groups and patients in different clinical conditions [5,6,7,8,9,10]. On the other hand, it is known that when an individual performs physical exertion during physical activity (PA), physiological alterations occur, such as increased ventilation and airflow velocity. These alterations hinder the filtering mechanism of existing pollutants in the air, normally performed by the patient’s nose, so that the majority of breathing becomes through the mouth [11]. This can lead to the transport of pollutants to the deepest region of the respiratory system, with harmful consequences for health [12]. In addition, during the natural process of aging, several comorbidities, such as obesity, hypertension, atherosclerosis, diabetes, cardiovascular diseases, and neurodegenerative diseases [13,14,15,16,17], can be aggravated by exposure to atmospheric pollutants.

In this sense, a broad review is necessary, analyzing the vast existing literature on the effects of air pollution on the health of older adults during PA. Mapping reviews are studies with high scientific impact, mapping and characterizing research, and identifying gaps in the literature in several areas [18,19]. This type of work is lacking in environmental sciences [18], specifically when considering the health of older adults during PA practices.

Although the adverse health effects of indoor or outdoor air pollution have been widely documented [20,21,22], and it is known to be a major cause of public and environmental health problems worldwide [18,23,24], little has been reported on the impacts that air pollutants can cause on the health of older adults during physical activity. Thus, in order to concentrate and analyze the vast amount of research available, we carried out a mapping review with the objective of analyzing the state of the art on the effects of air pollution on the health of older adults during physical activities.

## 2. Research Questions of the Review

On the basis of the work of Fernández-Sotos et al. [25], a key methodological aspect for a successful mapping review is the definition of research questions (RQs) to be answered. For this mapping review, we defined five RQs:

- **RQ1.** How many articles have been published about the effects of air pollution on the health of older adults during physical activity? What are the characteristics of the studies? What is the geographical distribution of the research carried out?
- **RQ2.** What were the most commonly investigated health-related outcomes?
- **RQ3.** Which air and environmental pollutants were the most commonly investigated?
- **RQ4.** Which types of PA were most investigated? Which ones presented greater health risks and which ones presented greater benefits when older adults were exposed to pollutants during PA practice? Which atmospheric and environmental pollutants compromised the health of older adults during PA practices?
- **RQ5.** What are the effects of air pollution on health outcomes during PA in older adults?

## 3. Methods

### 3.1. Guidelines

This mapping review was carried out to analyze and summarize the effects of air pollution on the health of older adults during PA. The work was based on the recommendations for systematic mapping in environmental sciences by James et al. [26].

Three filters were applied in this mapping review to gather (research procedure), select (inclusion and exclusion criteria), and extract (data extraction) relevant information from the literature [27]. The steps described in the Template for a Mapping Study protocol [28] were followed (see Table S1).

### 3.2. Search Strategy

A search for publications in English was performed in the PubMed, Web of Science, Scopus, and Cinahl databases. The search was performed using the descriptors summarized in Table 1. The final literature search was completed on 12 June 2022.

The Web of Science database was prioritized in duplicate article decisions, and searches were performed in the Core Collection, with terms related to older adults, air pollution, and exercise for the topic item, and the stipulated time as every year. See Table S2 in the supplemental material to view the search strategy used in the PubMed database.

### 3.3. Eligibility Criteria of the Studies

Two reviewers (A.D’O. and L.C.S.) independently performed the search and assessed the eligibility of each article. Discrepancies were resolved by a third researcher (A.A.). Only articles that investigated the effects of air pollution on the health of older adults during PA (through cohort studies; cross-sectional and longitudinal studies; and randomized, non-randomized, and quasi-randomized studies) with abstract and full text available online until June 12 from 2022 were selected. Studies with only abstracts available were not included. There were no restrictions on the date of publication to verify the evolution of scientific production.

Eligibility criteria for this mapping review were based on the Population, Exposure, Comparator, Outcome, Study Design (PECOS) statement [29] (Table 2).

### 3.4. Data Extraction and Analysis

The authors (A.D’O. and L.C.S.) independently extracted data from all studies and included them in a Microsoft Excel spreadsheet. For the analysis and discussion of the results, the following data were extracted: year of publication of the research, journal, country, and city (place of study), study design, sample characteristics (population, number of subjects, age and sex of participants), internal or external environment, pollutants, physical activity investigated, health outcomes, and conclusions.

The pollutants investigated were categorized according to Sun and Zhu [22]: (a) general gaseous pollutants (including ozone (O_3_), sulfur dioxide (SO_2_), carbon monoxide (CO), nitrogen dioxide (NO_2_)); (b) particulate matter (total suspended particles (TSP), particulate matter in suspension, PM_2.5_, PM_10_); and (c) other hazardous substances (toxic air pollutants, volatile organic pollutants, nitrogen oxides (NOx)).

The classification of health outcomes was based on the work of Sun and Zhu [22]: (1) respiratory diseases (asthma, respiratory infections, respiratory disorders, chronic obstructive pulmonary disease); (2) chronic diseases (diabetes, chronic respiratory diseases); (3) cardiovascular diseases (hypertension, heart rate variability, heart attack, cardiopulmonary disease, ischemic heart disease, blood clotting, deep vein thrombosis, stroke); (4) health records (morbidity, hospital admissions, outpatient consultations, emergency care, mortality); (5) cancer (bladder cancer, brain tumor, breast cancer, liver cancer, lung cancer, cancer (unspecified)); (6) mental disorders (Alzheimer’s disease, Parkinson’s disease, depression and stress, boredom, autism spectrum disorder, cognitive function, mental (behavioral) disorder); (7) other diseases (DNA methylation alterations, neurobehavioral functions, inflammatory diseases, skin diseases, deficiency); and (8) general health outcomes (health outcomes, such as muscle strength, functional capacity, inflammatory factors, etc., for older adults without a specific diagnosis, such as cardiovascular disease or cancer). Considering the results of the health outcomes of older adults during physical activities, the data were categorized according to the work of Sun and Zhu [22] into three parts: positive, negative, and ambiguous. In other words, studies that concluded that air pollution has harmful health effects during physical activities were classified as positive; those without harmful effects as negative; and, if there was more than one interpretation or limited evidence reported by the authors, the study was classified as ambiguous.

### 3.5. Study Quality Assessment

To investigate the risk of bias in the studies, we used the Mixed Methods Appraisal Tool (MMAT), version 2018 [30]. The reliability and validity of the new version of the MMAT have been confirmed [30,31]. The MMAT is designed for the appraisal of mixed study reviews, which include studies with qualitative, quantitative, and mixed methods. To assess the quality of studies, the MMAT contains two initial screening questions for all study types, followed by five questions for each of five possible types of study design. We used the same evaluation criteria with this tool adapted from May et al. [32]. The “Can’t tell” response category indicates that the article does not report information appropriate to answer “Yes” or “No”, or that it reports unclear information related to the criterion. “Yes” response categories indicate that the article reports appropriate information for the criterion, while “No” response categories indicate that the article does not report appropriate information for the criterion.

## 4. Results

### 4.1. Literature Search Results

**Figure 1:**
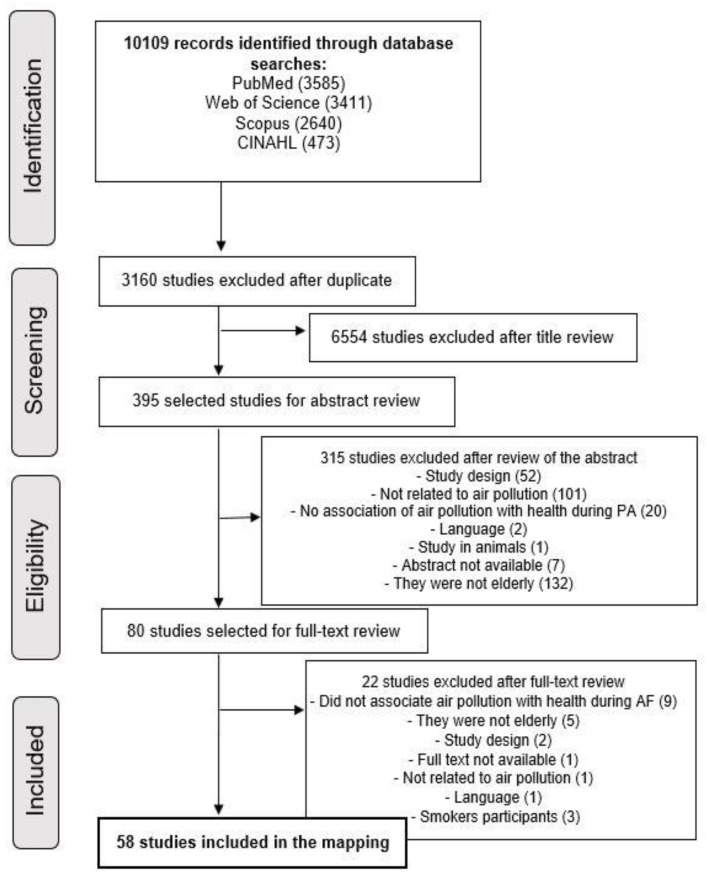
PRISMA flowchart illustrating the literature search and selection process.

The literature search identified 10,109 relevant articles. Of these, 80 full articles were evaluated for eligibility and 58 studies were selected for synthesis (Figure 1).

Below, the five research questions are answered on the basis of the synthesis of the analyses.

**RQ1**. How many articles have been published about the effects of air pollution on the health of older adults during physical activity? What are the characteristics of the studies? What is the geographical distribution of the research carried out?

**Figure 2:**
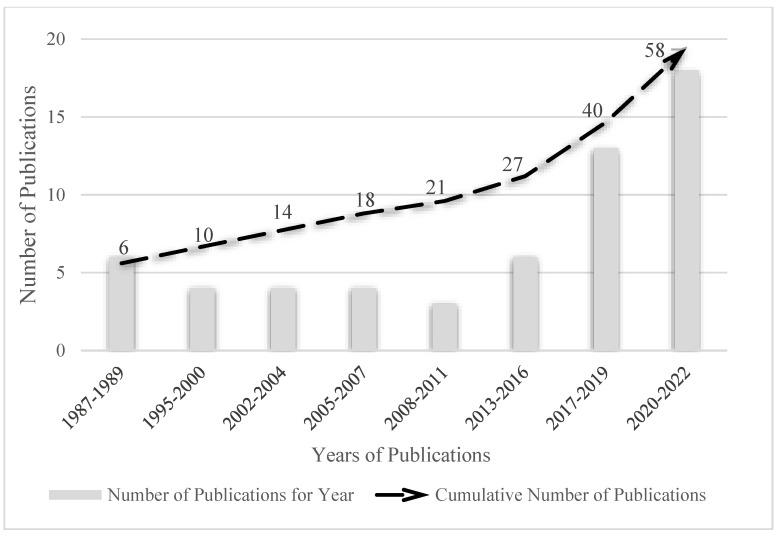
Number of articles every three years on the effects of air pollution on the health of older adults during physical activities.

The oldest study was published in 1987 [33], with a subsequent 58 published studies that investigated the topic by the year 2022 (see Table S3 of the Supplementary Material for a complete list of the selected studies). The period with the highest number of publications was between 2017 and 2019, with 13 studies, and 2020 to 2022, with 18 studies (Figure 2). The study with the longest follow-up period and duration was that of Elliot et al. [34], which lasted from 1988 to 2008.

**Figure 3:**
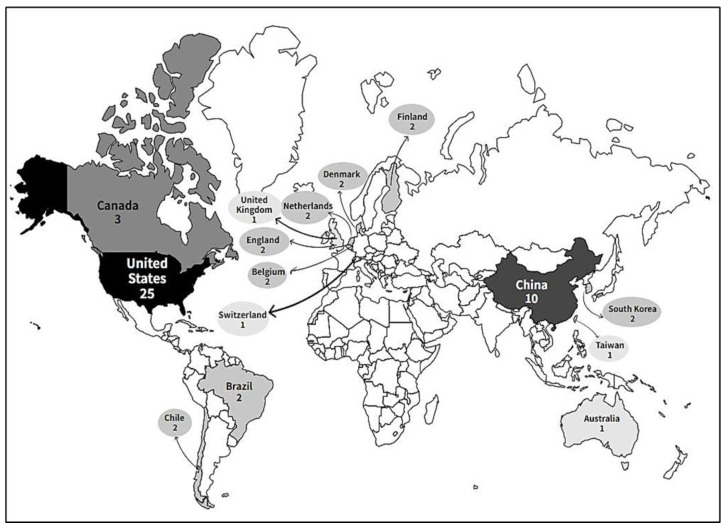
Global geographic distribution of research on the effects of air pollution on the health of older adults during physical activity.

The predominant countries that carried out the research were the USA, with 25 studies, followed by China with 10, and Canada with 3. Brazil, Chile, the Netherlands, Finland, England, Denmark, Belgium, and Korea performed two studies each, and Switzerland, Taiwan, United Kingdom, and Australia one study each (Figure 3).

Of the 58 included studies, 20 had a longitudinal methodological design and 15 carried out a longitudinal experimental model. The remainder were classified as cross-sectional (six studies); randomized controlled trials (5); pre- and post-analysis (3); longitudinal prospective cohort studies (2); repeated measures studies (2); experimental (2); and one each of cross-sectional retrospective, longitudinal prospective, and quasi-experimental prospective.

The studies included a total of 1,653,411 participants. The study with the largest sample was by Kim et al. [35] with 1,259,871 participants, in a longitudinal study. The studies with the smallest number of participants were those of Babb [36] and Drechsler-Parks [37], both experimental studies. The majority of the studies had a sample composed of men and women, totaling 51 studies. In three studies, only men participated, and in another three, only women; one study did not report these data. The average age of the participants varied, with the study with the youngest sample being by Huang et al. [38], aged 50 years or older. The study with the oldest sample was carried out by Bagheri et al. [39], including participants aged between 65 and 90 years or older. In the majority of studies, 28 participants had a mean age of 64 years (age range from 60 to 79 years).

Of the included studies, 23 involved older people without specificity in relation to health; 8 included healthy older adults and non-smokers; 4 only healthy older people, without specifying tobacco use or not; and 3 included non-smoking older people, not necessarily healthy. Some studies included older adults with specific diagnoses, six with a diagnosis of coronary artery disease (CAD) and non-smokers, one study with a diagnosis of CAD (without specifying tobacco use or not), and three studies included participants with a diagnosis of COPD. Only the study by Babb et al. [40] exclusively investigated older people who regularly exercise.

**RQ2.** What were the most commonly investigated health-related outcomes?

Several health outcomes were investigated. The most common were cardiovascular disease outcomes, investigated in 14 studies in isolation and in another 10 together with other outcomes. The outcomes related to cardiovascular diseases mainly included blood pressure, a decline in the ST segment on the electrocardiogram (associated with myocardial ischemia), and heart rate. The second most investigated outcome was respiratory, in 19 studies (9 studies exclusively respiratory outcomes and another 10 combined). Among these outcomes, pulmonary function and peripheral oxygenation level stood out.

Outcomes of mental disorders were evaluated in 11 studies, with depression and cognition being the most present. Overall health outcomes were evaluated in nine publications, including outcomes such as upper and lower limb muscle strength, hand grip strength, sedentary behavior, and time outdoors. Five articles reported health records of older adults, including mortality and hospitalization data. Only three articles reported outcomes considered to be chronic diseases, in these cases, diabetes. Two articles explored other diseases, with outcomes of oxidative stress markers and liver function levels. No studies selected in the mapping review investigated cancer.

**RQ3.** Which air and environmental pollutants were most commonly investigated? What is the relationship between health outcomes and air pollutants during PA in older adults?

The investigated environments were classified into two types: internal or external. External environments, such as squares and parks, were present in 42 studies. Indoor environments were used in 15 studies, including research laboratories [41], with a controlled level of exposure to the pollutant, such as pollutant gases, except for the study by Gong et al. [42] who also controlled particle level. Regarding the analyzed area, 43 studies investigated urban areas, 4 investigated rural areas (only one exclusively), and 1 investigated commercial areas. These data were not reported in 15 studies.

Considering the class of pollutants, fine particles were the most widely investigated among the analyzed studies; PM_2.5_ was analyzed in 35 studies, and in 10 of these, it was the only pollutant. Among fine particles, PM_10_ was analyzed in 12 studies, PM_1.0_ in 2, and PM_4.0_ and PM*_coarse_* in 1 study each; three studies analyzed the level of fine particles, and two studies the total number of particles.

The second most investigated class was pollutant gases, with the most frequently investigated being NO_2_ and O_3_ in 24 studies each, followed by CO in 14 studies, SO_2_ in 12 studies, and CO_2_ and/He-O_2_ in two studies. Other substances and biomasses were investigated in the fewest studies; NO_x_ was evaluated in four publications, black carbon in five, organic carbon in three, and elemental carbon in two studies.

Regarding the results of health outcomes and pollutants during PA in older adults, it can be seen that the most investigated pollutants were PM_2_._5_ and PM_10_, present in all health outcomes. The most commonly investigated relationship between air pollutants and health outcomes was the effects of PM_2.5_ on cardiovascular disease (18 studies). Table 3 shows the number of studies according to air pollutants and the health outcomes of the older adults during PA.

**RQ4.** Which PAs were most commonly investigated? Which presented greater health benefits, and which presented greater risks when the older adults were exposed to pollutants during PA practice? Which atmospheric and environmental pollutants compromised the health of the older adults during PA practices?

Of the 58 articles that entered the mapping review, 26 of the authors did not report the PA that the older adults performed. Walking was investigated in 17 studies, followed by cycle ergometer exercise in 9 studies. Varied PA, such as playing ball, walking uphill, swimming, cycling, running, slow walking, stretching, and traditional Chinese exercises were investigated in seven studies, cycling in three studies, and the practice of resistance training with weights in only one study. Twenty-nine studies carried out specific programs or protocols in the investigated PA.

In 20 studies, PA was measured from the time and total days (minutes/week) performed, in 15 through various components such as distance, telemetry, time in minutes, heart rate, accelerometer, speed by GPS, electrocardiogram, arterial saturation of oxygen by pulse oximetry, blood pressure, perceived exertion rate, ventilation rate by plethysmography, and gas exchange measurements. In nine studies, PA was measured from the ventilatory volume per minute through the respiratory rate and in another four through the intensity of each type of PA attributed to the standard metabolic equivalent of the task value (MET-h/wk). In two studies, arterial oxygen saturation was measured by pulse oximetry and intensity measurement through actigraph, and a multisensory activity monitor was used for the average steps per day. In two studies, only one questionnaire was used to gather information on whether or not the participants performed PA, one study each used the one-repetition maximum strength test or 6 min walk test to measure PA, and only one did not report the way that PA was measured.

The following tables show the PAs, sample characteristics (sex and age), pollutants, health outcomes, and findings on benefits (grouped as negative) (Table 4); risks (grouped as positive) (Table 5); and those which presented limited interpretation or evidence (grouped as ambiguous) (Table 6) of PA on health outcomes of older adults during exposure to polluted environments.

Of the 58 studies investigated, in 18, the PA showed benefits to the health of the older adults during exposure to pollutants. The most frequently reported PAs were PA in general (four studies), followed by PA in leisure, leisure and transport, moderate to vigorous, and slow walking accompanied by other activities in three studies each. Walking only, a controlled exercise protocol (stretching, treadmill walking, park walking), incremental cycle ergometer test, and resistance training were employed in only one study each. Urban environments were the most frequently (12 studies) investigated during these PA, followed by five studies that did not report the environment and one with urban and rural environments. With respect to open and closed environments, 16 studies were carried out in open environments, 1 in a closed environment, and 1 in a mixed (open and closed) environment. As for air pollutants that did not compromise the health of older adults during PA practices, PM_2.5_ was the most frequent, in 14 studies, followed by NO_2_ and PM_10_ in seven studies (Table 4).

In 22 studies, the exposure of the older adults to pollutants presented health risks during PA, more frequently to PA in general (seven studies), followed by submaximal exercise tests on a cycle ergometer and exercise bike in five studies, walking outdoors and PA outdoors in three, and cycle ergometer and incremental exercise on a treadmill in two studies each. The environments by location investigated during these PA were urban in 19 studies, rural in 1 study, and not reported in 2 studies. Open environments were investigated in 16 studies and closed environments in 6 studies. PM_2.5_ was the air pollutant that presented the greatest risk to older adults during PA in 13 studies, followed by O_3_ in 11, CO in 8, and NO_2_ in 6; other pollutants and information about these studies can be found in Table 5.

**RQ5.** What are the effects of air pollution on health outcomes during PA in older adults?

**Figure 4:**
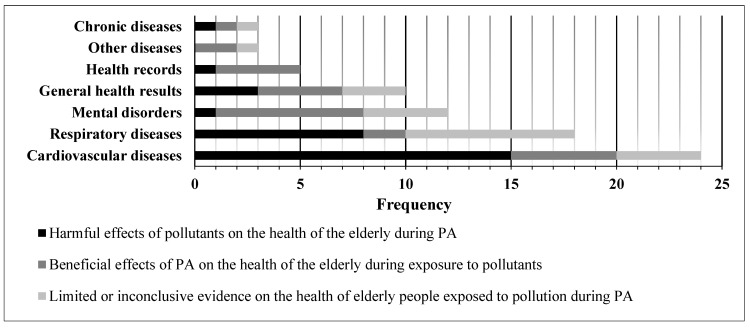
Number of studies according to the health outcomes investigated during physical activity in older adults and the effects of air pollution.

Regarding the health outcomes investigated in the older adults, for the effects of air pollution during PA, of the 75 outcomes investigated, air pollution had harmful effects on the health of the older adults during PA practice in 29, more frequently in cardiovascular diseases. On the other hand, in 25 outcomes, the beneficial effects of PA on the health of the older adults remained despite exposure to high and low concentrations of pollutants, more frequently in mental disorders; and in 21, the evidence was limited or inconclusive, especially in respiratory diseases. Figure 4 presents the frequency according to the health outcomes investigated during PA in the older adults and the effects of pollution.

### 4.2. Risk of Bias Assessment

Table 7 presents the results of the quality assessment. In total, 14 of the 58 studies that entered the present review did not have any negative classification, indicating high quality. A further 11 articles had only one negative classification and 14 two negative ratings, presenting medium-to-moderate quality. However, 13 studies presented between four and five negative classifications, indicating low quality. This factor occurred mainly due to the lack of methodological detail in the studies.

## 5. Discussion

This is the first mapping review carried out on the effects of air pollution on the health of older adults during PA practices, considering articles with different designs. Air pollution is one of the greatest environmental threats to human health [3], while PA practices present several health benefits [90]. Harmful, beneficial, and inconclusive effects on the health outcomes of older adults during PA and exposure to air pollution were observed in the studies; thus, we sought to develop this discussion on the basis of these results and relate it to the various findings on this topic in the literature.

The benefits of PA and exercise for older adults are widely known and documented in several health outcomes, such as cardiovascular, respiratory, cognitive, psychological, and quality of life [91]. In the present review, 16 studies showed that the benefits of PA were maintained even when considering exposure to air pollutants. Mental disorders was the outcome with the highest amount of evidence of benefits during exposure to pollutants, without studies pointing out risks. Currently, the deleterious effects of air pollution on mental and cognitive health are well established, for example, it is known that exposure to PM_2.5_ and NO_2_ is a risk factor for the development of Alzheimer’s disease and that increased exposure to PM_2.5_, NO_2_, and O_3_ is related to the development of depression in older adults [92,93,94,95,96]. In contrast, exercise and PA in general produce effects that decrease depression and improve mood in older adults [97,98], as well as being a recognized way to treat Alzheimer’s disease [5]. Thus, the findings demonstrate the importance of PA in the mental health of the older population.

PA has been recommended for older people with cardiovascular disease, and studies are well established on this topic in the literature [99]. However, on the basis of the articles included investigating cardiovascular diseases, we observed that the practice of PA during exposure to pollutants can further compromise the conditions of this disease. On the other hand, it is important to note that of the 15 studies that reported these harmful results, in 8 of them, the participants already had cardiovascular diseases [42,63,64,65,66] or a high prevalence of developing cardiovascular diseases [70,72], while in the 5 studies that reported beneficial results, the sample was made up of healthy older adults, without a specific diagnosis. This demonstrates that PA even under exposure to air pollutants can provide a mechanism for preventing and maintaining cardiovascular health in older adults, as shown in the studies by Elliott et al. [34], Endes et al. [53], and Kubesch et al. [52] who demonstrated the benefits of PA in relation to the risk of myocardial infarction, stroke, and arterial stiffness.

The literature already points out that the adverse effects of air pollution can potentiate comorbidities, such as preexisting cardiovascular diseases, even attenuating the beneficial effects of PA on practitioners [100]. However, the population-based study by Kim et al. [35] (189,771 participants, more than 50% of whom were older adults) with a two-year follow-up found that moderate-to-vigorous PA ≥5 times/week was significantly associated with a decrease in the risk of cardiovascular disease, even when exposed to low and high levels of PM_10._ These results were also maintained during exposure to high and low levels of PM_2,5_, except for coronary heart disease [35].

However, air pollution had a negative impact on healthy older people during PA, both in cardiovascular and respiratory health variables, presenting acute adverse symptoms in the study by Stieb et al. [58,60], including pulmonary vascular and inflammatory changes [68], as well as increased heart rate [61]. These adverse symptoms found in older adults can be justified by the direct inhalation of environmental gases, or by oxidative stress due to exposure to various pollutants [61].

Of the studies that only investigated respiratory diseases, the results are divided as to the risks of the practice of PA by older adults in environments with air pollution, presenting limited or inconclusive evidence in eight studies each. Despite this result, two studies showed benefits from the practice despite the exposure [40,50], both being longitudinal experimental studies. These results are controversial considering those published in the literature, primarily because most of the participants in these two studies are older women [101,102], and secondly because high ventilation induced by PA increases the exposure and rate of particle deposition in the lungs [103,104], which potentiates harmful effects on health [12]. In addition, high-quality evidence from a recent systematic review of randomized controlled trials indicated that moderate-to-vigorous PA practice during exposure to pollutants has adverse effects on lung function and related symptoms [105].

Considering health records, we observed that the majority of studies that investigated this outcome maintained the beneficial effects of PA on the health of the older adults during exposure to pollutants [34,47,54,56]. This is in contrast to recent literature results and the WHO itself, since exposure to pollutants is strongly associated with the risk of disease development and mortality prevalence, for example, long-term exposure to PM_2.5_ or NO_2_ is associated with stroke, NO_2_ is associated with higher cases of atrial fibrillation, and exposure to O_3_ increases the risk of hospitalizations for pneumonia and COPD exacerbations [3,106,107]. On the other hand, PA practice promotes the opposite outcome. The study by Cunningham et al. [91] found a reduction in the risk of CAD in men and a 40% reduction in mortality from cardiovascular diseases in moderate-to-vigorous PA practitioners. In the current review, PA practice provided lower cardiovascular and respiratory mortality and mortality in general [47,54,56], even with exposure to air pollutants. In the study by Yu et al. [59], it was found that exposure to PM_2,5_ contributed to the reduction in PA levels in older adults. In addition, a negative association was found between PA score and leisure time with days of hospital care in one year. Although the findings do not indicate cause and effect, this demonstrates that air pollution indirectly affects the health of older adults, as it reduces the practice of PA, which is associated with hospitalization.

Few studies have analyzed the effects of exposure to pollution during PA on chronic or other diseases, in addition to respiratory, cardiovascular, and mental disorders in older adults. Of the four studies that analyzed these effects, none presented risks to the health of the older adults. The study by Kim et al. [35] points out that moderate-to-vigorous PA decreases the risk of developing diabetes, even with exposure to pollutants, which is extremely important, as currently 25% of older adults in the USA have a diagnosis of diabetes and 48% are pre-diabetic [108].

The study by Cassilhas et al. [41] reported an important comparison of PA in an open environment versus a closed environment, and although the practice of PA in an open environment does not produce risks, when the same activity is performed in a closed environment, it has greater benefits for other diseases and cognition of older adults. Unfortunately, none of the studies included in the review analyzed the outcome of cancer. However, in the current literature, there is strong evidence that pollution and high levels of PM increase the incidence and mortality of lung cancer, although for other cancers, the evidence is limited [109].

### 5.1. Limitations and Future Studies

Although 53 studies have been published since 1987, focusing on the last few years, an effort by researchers is necessary to produce knowledge about the effects of air pollution on the health of older adults during PA, because despite the relevant number of published studies, the results are divergent, limiting the development of specific conclusions. Most of the studies also did not report the lifestyle, habits, where they worked, and the environments where the participants lived in recent years and decades, which, if they were environments with high levels of pollution, could affect the results of the research in which they participated. The focus of further studies should be on evaluating different models and scenarios, combining types of PA in different environments in relation to different levels of air pollution, and calculating possible risks to the health of older adults. Knowing the limits older adults can be exposed to when they decide to practice indoor and outdoor PA is essential, in addition to developing safety recommendations for this population.

### 5.2. Innovations, Study Strengths, and Practical Applications

To our knowledge, after an extensive search in international databases, the current mapping review is the first to be developed in the literature. In addition to this innovative and relevant character, considering that the older population is highly sensitive to air pollution, this review is even more relevant because this sensitivity is enhanced when they are practicing PA.

In the current scenario of the COVID-19 pandemic, the need for studies with these outcomes increases even more [110,111]. The idea is to provide greater safety and evidence of the risks and benefits to older adults, in different scenarios of pollution, environments, and PA.

Safety recommendations should be analyzed and written for the practice of PA by older adults in different environments, on the basis of the data of this mapping review and other systematic reviews under development. The results and our analyses and conclusions allow several practical applications, including greater attention to the previous health conditions of older adults in relation to PA practice environments, taking into account previous diseases, for example, heart diseases, in relation to environments polluted with nanoparticles from the combustion of organic material, which can significantly increase cardiorespiratory health risks.

On the basis of the recommendations of the studies included in this mapping review, we stratified relevant information for public policymakers, health professionals, researchers, and practitioners of physical activities, in order to provide guidance on the topic addressed in this review, and from there, to develop new public policies based on the evidence presented, as well as identifying gaps to be addressed in further research. Guidelines for older practitioners of physical activities in environments with levels of pollution were highlighted. On the basis of these points, care and observations that need to be taken into account for PA practice are presented in Table 8.

## 6. Conclusions

On the basis of the current mapping review of 58 studies from four databases to analyze the state of the art on the effects of air pollution on the health of older adults during physical activities, we concluded that air pollution and poor air quality are harmful factors to the health of older adults during the practice of PAs. A significant number of studies show that air pollution has harmful effects on the health of older adults during the practice of PA, with greater frequencies in cardiovascular and respiratory diseases. On the other hand, in mental-health-related outcomes (depression and cognition), the beneficial effects of PA in older adults were maintained even during exposure to pollutants at their highest frequency. The most investigated pollutants during PA in the older adults were PM_2.5_ and PM_10_, and the most commonly investigated relationship between air pollutants and health outcomes was on the effects of PM_2.5_ in cardiovascular disease. Walking was the most widely investigated PA, followed by cycle ergometer exercise, and PA such as playing ball, walking uphill, swimming, cycling, running, slow walking, stretching, and traditional Chinese exercises, in addition to resistance training with weights. Future studies should combine different types of PA in several environments, considering both indoor and outdoor pollutants to calculate risks to the health of older adults.

## Figures and Tables

**Table 1:** Search strategy adopted for the mapping review.

Search Terms	Descriptors
1. Elderly	Elderly OR Aged OR Aging OR “Aged, 80 and over” OR “older adults” OR “older women” OR “older men” OR Senescence OR “Oldest Old” OR “Old Adults” OR Nonagenarian* OR Octogenarian* OR Centenarian *
2. Air pollution	“air pollution” OR “air pollutant*” OR “air quality” OR “particulate matter” OR “PM_10_” OR “PM_2.5_” OR “carbon monoxide” OR “carbon dioxide” OR “ozone” OR “nitrogen dioxide” OR “sulfur dioxide” OR “traffic-related air pollution”
3. Exercise	“exercise” OR “physical exercise*” OR “physical activity” OR “physical exertion” OR “physical training” OR “sport*” OR “resistance training” OR “strength training” OR “aerobic exercise” OR “aerobic fitness” OR “athlete” OR “athletic performance” OR “running” OR “cycling”
Combination	#1 AND #2 AND #3

**Table 2:** Criteria for inclusion and exclusion of studies selected for review.

		Inclusion Criteria	Exclusion Criteria
P	Participate	Healthy and sick older adults	
E	Exposure	Air pollution (particulates, carbon monoxide, carbon dioxide, ozone, nitrogen dioxide, sulfur dioxide)	Temperature, secondhand smoke, PM chemical constituents and acute intoxications
C	Comparison	-	-
O	Outcome	Effects of air pollution on the health of older adults during physical activities	-
S	Study	Cohort, cross-sectional, longitudinal and randomized, non-randomized and quasi-randomized clinical trials	Reviews, meta-analyses, case study, comments, letters, and editorials

**Table 3:** Number of studies according to air pollutants and health outcomes of older adults during physical activities.

Health Outcomes during PA	Air Pollutants									
	Gaseous Pollutants					Particulate Material			Other Hazardous Substances	
	O_3_	SO_2_	CO	NO_2_	HE-O_2_	PM_2.5_	PM_10_	TSP	NOx	VOPs
Respiratory diseases	11	7	6	8	2	7	2	-	-	2
Cardiovascular diseases	12	7	13	14	1	18	5	4	3	11
Health records	1	1	-	2	-	3	1	-	-	-
General health results	4	3	2	5	-	7	3	1	1	-
Mental disorders	2	2	-	4	-	10	4	3	-	-
Other diseases	2	1	1	1	-	2	1	3	-	-
Chronic diseases	1	-	-	-	-	2	2	1	-	-
Total	33	21	22	34	3	49	18	12	4	13

**Table 4:** Sample characteristics (sex and age), pollutants, physical activity, health outcomes, and findings on the beneficial effects of PA on the health of older adults during exposure to polluted environments.

Authors, Year	Country/City	Positive, Negative, and Ambiguous	Population	Age $(\bar{x\;}$ ± SD/Min-Max)	Sex	Health Outcomes	PA Type	Exposure (Pollutants)	Environment-Open/Closed	Environment by Location (Urban/Rural/Commercial)	Main Findings of the Benefits of PA in Polluted Environments
Liu et al. (2022) [43]	China/NR	Negative	Residents of urban areas	63.99 ± 9.57	♀ ♂	General health results	Physical activity in transport and leisure	PM_2.5_	Open	Urban	Green spaces indirectly encourage more PA, reducing PM_2.5_ concentrations, thus contributing to better subjective well-being.
Yao et al. (2022) [44]	China/NR	Negative	Participants from county-level communities of provinces in China	60.90 ± 9.25	♀ ♂	Mental disorders	PA in general	PM_2.5_	Open	Rural/urban	Lower levels of PA increased the impact of environmental PM_2.5_ on depressive symptoms.
Wu et al. (2022) [45]	UK/NR	Negative	Older adults with and without depressive disorders	64.1/NR	♀ ♂	Mental disorders and general health outcomes	Walking and MVPA	PM_2.5_ and PM_10_	Open	Urban	Prolonged exposure to PM_2.5_ and low levels of vitamin D and physical activity were associated with an increased risk of major depressive disorder. Furthermore, high levels of vitamin D and physical activity may attenuate the relationship between PM_2.5_ and major depressive disorder in older adults.
Cassilhas et al. (2022) [41]	Brazil/São Paulo	Negative	Sedentary older adults	67.85 ± 2.34	NR	Mental disorders and other illnesses	Exercise protocol for the groups: Control (stretching), Internal Exercises (walking on a treadmill), and External Exercises (walking in the park)	UFP, PM_1.0_, PM_2.5_, PM_4.0_, and PM_10_	Open close	Urban	Indoor walking was shown to provide better cognitive and physiological outcomes than outdoor exercise.
Ran et al. (2021) [46]	China/Hong Kong	Negative	Seniors	65/NR	♀ ♂	Mental disorders	Slow walking, stretching, traditional Chinese (Luk Tung Kuen, Tuen Kam, and Tai Chi), aerobic exercise (playing ball, walking uphill, swimming, cycling, and running)	PM_2.5_	Open	Urban	The benefits of PA remain in people exposed to various levels of PM_2,5_, and it is recommended for people living in relatively polluted areas.
Kim et al. (2020) [35]	South Korea/Seoul, Busan, and Incheon	Negative	Older people over 50 with a history of diabetes	64.7 ± 6.2	♀ ♂	Chronic diseases	MVPA	PM_10_ and PM_2.5_	Open	Urban	The increase in MVPA appeared to reduce the risk of diabetes in groups with high and low/moderate levels of exposure to PM_10_ or PM_2.5_ in older adults.
Sun et al. (2020) [47]	China/Hong Kong	Negative	Seniors	71.9 ± NR	♀ ♂	Health records	Slow walking, stretching, traditional Chinese exercises (Tai Chi, Pak Tuen Kam, and Luk Tung Kuen), aerobic exercise (running, cycling, swimming, walking uphill, and playing ball)	PM_2.5_	Open	NR	Moderate-to-high volume of PA was associated with a lower risk of cardiovascular and respiratory mortality. PA can still be recommended for older people living in relatively more polluted areas.
Molina- Sotomayor et al. (2020) [48]	Chile/Santiago and Vinã del mar	Negative	Sedentary older adults	70.1 ± 5.3	♀	Mental disorders and general health outcomes	Resistance training	PM_10_, PM_2.5_, NO_2_, O_3_, and SO_2_	Open	Urban	Resistance training, even in an environment with a high concentration of pollutants, promoted benefits in the concentration of IGF-1, strength, and cognition of the older adults.
Elliott et al. (2020) [34]	USA/NR	Negative	Older people and American adult nurses	63.1 ± 8.9	♀	Cardiovascular disease and health records	PA at leisure	PM_2.5_	Open	NR	PA was beneficial for the risk of general cardiovascular disease, myocardial infarction, stroke, and general mortality at environmental PM_2.5_ levels.
Wang et al. (2020) [49]	China/NR	Negative	Older adults	67.2 ± 6.3	♀ ♂	Mental disorders	General PA	PM_2.5_	Open	NR	The relationship between PM_2.5_ and depression was mediated by outdoor PA. The relationship between depression and PA weakened with increasing PM_2.5_ level.
Molina-Sotomayor et al. (2019) [50]	Chile/Santiago and Vinã del mar	Negative	Older adults	68.9 ± 2.85	♀	Mental disorders, cardiovascular and respiratory diseases	Walk	PM_10_, PM_2.5_, NO_2_, O_3_, and SO_2_	Open	Urban	Planned, regular, and systematic cardiorespiratory PA had a positive influence on brain integrity and oxygen transport mechanisms, regardless of air pollution levels.
Chen et al. (2019) [51]	Taiwan/Taipei	Negative	Seniors	70.21 ± 3.92	♀ ♂	General health results	General PA	PM_2.5_, NO_2_, CO, O_3_, and SO_2_	Open	Urban	The level of PA modified the effect of PM_2.5_ on the body composition of the older adults, lower levels of physical activity were associated with greater loss of muscle mass, and increase in fat mass was related to exposure to the pollutant.
Kubesch et al. (2018) [52]	Denmark/Copenhagen and Aarhus	Negative	Cancer-free seniors	56.7 ± 4.4	♀ ♂	Cardiovascular diseases	AF in leisure and transport	NO_2_	Open	Urban	The long-term benefits of PA in preventing the development of infarction in healthy older adults, and possibly as effective control of the disease in patients with previous infarction, may outweigh the risks associated with increased residential exposure to traffic-related air pollution during PA.
Endes et al. (2017) [53]	Switzerland/NR	Negative	Older adults and seniors	63.6 ± 8.2	♀ ♂	Cardiovascular diseases	AFMV	PM_10_, PM_2.5_, and NO_2_	Open	NR	The probability of having a higher level of arterial stiffness was significantly higher with higher levels of exposure to pollutants for inactive individuals, but not for active individuals. Moderate-to-vigorous physical activity confers a protective effect against the adverse vascular effects of air pollution in low-pollution environments.
Andersen et al. (2015) [54]	Denmark/Copenhagen and Aarhus	Negative	Older adults without a previous diagnosis of cancer at baseline	56.6 ± 4.3	♀ ♂	Health records	AF in leisure and transport—participation in sports, cycling, walking, and gardening	NO_2_	Open	Urban	Exposure to high levels of NO_2_ did not modify the inverse associations between cycling, gardening, and mortality, indicating beneficial effects of these activities on mortality.
Kim et al. (2015) [55]	Korea/Seoul	Negative	Seniors	70.6 ± NR	♀ ♂	Other diseases	Physical exercise	PM_2.5_, NO_2_, O_3_, CO, and SO_2_	Open	Urban	The adverse effects of pollution on liver enzyme levels can be reduced through regular exercise.
Wong et al. (2007) [56]	China/Hong Kong	Negative	Chinese who died aged 30 or over	73.6 ± 13	♀ ♂	Health records	Exercise at leisure	NO_2_, SO_2_, PM_10_, and O_3_	Open	Urban	PA can help protect against air-pollution-induced mortality, as people who practice PA were at a lower risk of mortality from ambient air pollution than inactive people.
Babb et al. (2003) [40]	USA/Dallas	Negative	Senior runners who exercise regularly	70.5 ± 9.5	♀ ♂	Cardiovascular and respiratory diseases	Incremental test on cycle ergometer	CO_2_e and He-O_2_	Closed	NR	The ventilatory response to exercise of senior runners was adequate to support their increased exercise capacity, and physical training did not alter the ventilatory response to exercise with He-O_2_ or CO_2_e breathing and inspired breathing.

♀: female; ♂: male; PA: physical activity; MVPA: moderate-to-vigorous physical activity; CO: carbon monoxide; CO_2_e: carbon dioxide equivalent; DV: standard deviation; USA: United States of America; He-O_2_: helium-oxygen; Min.: minimum; Max: maximum; PM: particulate matter; NO_2_: nitrogen dioxide; NR: not reported; O_3_: ozone; SO_2_: sulfur dioxide; UFP: ultrafine particles; $\bar{x}$: medium.

**Table 5:** Sample characteristics (sex and age), pollutants, physical activity, health outcomes, and findings on the risks of pollutants on the health of older adults during PA.

Authors, Year	Country/City	Positive, Negative, and Ambiguous	Population	Age $(\bar{x\;}$ ± SD/Min-Max)	Sex	Health Outcomes	PA Type	Exposure (Pollutants)	Environment-Open/Closed	Environment by Location (Urban/Rural/Commercial)	Main Findings of the Risks of Exposure to Pollutants during AF
Yu et al. (2021) [15]	United States/Sacramento	Positive	Mexican American seniors	70.5 ± 6.9	♀ ♂	Chronic diseases	PA at leisure and outdoors	O_3_	Open	Urban	O_3_ exposure increased the risk of diabetes among older Mexican Americans, particularly among those with higher levels of leisure-time outdoor physical activity.
Bagheri et al. (2021) [39]	Australia/Adelaide	Positive	Australian seniors aged 65 and over	65 ≥ 90 *	♀ ♂	Mental disorders	General PA	NO_2_	Open	Urban	A one-standard deviation (1-SD) increase in NO_2_ and walkability score was associated with a 10% greater odds of any dementia versus no dementia.
Zwart et al. (2018) [57]	Netherlands/NR	Positive	Adults and older adults	75.5 ± 8.5	♀ ♂	General health results	AF in six categories—walking outdoors, cycling, doing light or heavy household activities, and a maximum of two sports	NO_2_, NO_x_, PM_2.5_, PM_10_, and PM*_coarse_*	Open	Urban	Exposure to air pollution can negatively affect the physical performance of older adults.
Stieb et al. (2018) [58]	Canada/Prince George	Positive	Non-smoking older adults	63.5 ± 6.5	♀ ♂	Cardiovascular and respiratory diseases	Light physical exercise outdoors in winter	CO, NO_2_, O_3_, PM_2.5_, and SO_2_	Open	Urban	Acute subclinical effects of air pollution were observed in older adults who exercise outdoors in winter.
Yu et al. (2017) [59]	China/Beijing	Positive	Retired older adults	66.63 ± NR	♀ ♂	General health outcomes and health records	General and leisure PA	PM_2.5_	Open	Urban	The increase in PM_2.5_ concentration was associated with a reduction in total walking hours, leisure PA, and total PA. The PA score was negatively associated with days spent in hospital. The higher the PA level, the fewer days in the hospital.
Stieb et al. (2017) [60]	Canada/Kincardine	Positive	Older adults, non-smokers, not exposed to tobacco in the home environment and without seasonal allergies	64 ± 5.5	♀ ♂	Cardiovascular and respiratory diseases	Light physical exercise outdoors in winter	CO, NO_2_, O_3_, PM_2.5_, and SO_2_	Open	Rural	Acute adverse cardiorespiratory effects were observed in rural older adults who exercise outdoors when air pollution levels are particularly high.
Salicio et al. (2016) [61]	Brazil/Cuiabá	Positive	Older adult exercisers, healthy and non-smokers	68.8 ± 4.7	♀ ♂	Cardiovascular and respiratory diseases	Physical exercise outdoors	CO and PM_2.5_	Open	Urban	Older people suffered environmental influences altering levels of exhaled CO, carboxyhemoglobin, and heart rate during exercise.
Alahmari et al. (2015) [62]	England/London	Positive	Older people with COPD and healthy individuals	71.1 ± 8.7	♀ ♂	General health results	General PA	PM_10_ and O_3_	Open	Urban	Increased O_3_ and PM_10_ exposure was associated with reduced physical activity levels in COPD patients.
Delfino et al. (2011) [63]	USA/Los Angeles	Positive	Retired older adults, diagnosed with CAD and non-smokers	65 years or older	♀ ♂	Cardiovascular diseases	General PA	PM_2.5_, organic carbon, elementary carbon, black carbon, O_3_, NO_2_, NO_x_, and CO	Open	Urban	The results suggest that the primary products of fossil fuel combustion carry an increased risk of myocardial ischemia. The AF measured by the actigraph was not able to change the findings of the effect of pollutants on the ST segment.
Delfino et al. (2010) [64]	USA/Los Angeles	Positive	Retired older adults, diagnosed with CAD and non-smokers	84 ± 5.6	♀ ♂	Cardiovascular diseases	General PA	Black carbon, organic carbon, PM_2.5_, NO_x_, CO, and O_3_ and total number of particles	Open	Urban	Moderate-to-strenuous exertion (daily and antigraph) was correlated with increased systolic and diastolic BP. AF was not associated with attenuation of the effects of pollution. There was a greater association between pollutants and increased PA in participants with obesity.
Lanki et al. (2008) [65]	Finland/Helsinki	Positive	Non-smoking seniors with coronary heart disease	68 ± 7	♀ ♂	Cardiovascular diseases	Submaximal exercise test on an exercise bike	PM_2.5_	Open	Urban	An increased risk of exercise-induced cardiac ischemia was observed in association with hourly variation in personal exposure to PM_2.5_ and outdoor levels in the 24 h prior to the exercise test.
Lanki et al. (2006) [66]	Finland/Helsinki	Positive	Older adult non-smokers and those diagnosed with CAD	68.2 ± 6.5	♀ ♂	Cardiovascular diseases	Submaximal exercise test on cycle ergometer	PM_2.5_	Open	Urban	The fraction of PM from combustion processes, notably from traffic, may be responsible for the effect observed in exercise-induced ischemic heart disease on days with high air pollution.
Gold et al. (2005) [67]	USA/Boston	Positive	Seniors	73 ± NR	♀ ♂	Cardiovascular diseases	Outdoor walk	PM_2.5_, black carbon, and CO	Open	Urban	ST-segment depression, possibly representing myocardial ischemia or inflammation, had stronger effects in the post-exercise periods associated with increased exposure to particles whose predominant source was traffic.
Gong et al. (2005) [42]	USA/Los Angeles	Positive	Older adults with and without COPD	70 ± 9	♀ ♂	Cardiovascular and respiratory diseases	Submaximal exercise	NO_2_ and particles concentrated in the environment (predominantly PM_2.5_)	Closed	Urban	Healthy older adults seemed more susceptible to urban fine particulate exposure during AF, presenting with acute small airway dysfunction with impaired gas exchange. This suggests that the respiratory effect may be related to the efficient penetration and deposition of inhaled toxic particles in the small distal airways.
DeMeo et al. (2004) [68]	USA/Massachusetts	Positive	Older volunteers	73.3 ± NR	♀ ♂	Cardiovascular and respiratory diseases	Outdoor walk	PM_2.5_, CO, O_3_, NO_2_, and SO_2_	Open	Urban	A statistically significant effect of ambient particulate air pollution on decreasing resting (post-exercise) oxygen saturation in an older adult population was demonstrated. Decreased oxygen saturation associated with air pollution may result from subtle particle-related pulmonary vascular and/or inflammatory changes.
Gong et al. (2004) [69]	USA/Los Angeles	Positive	Older adults with and without COPD	70.5 ± 9.5	♀ ♂	Cardiovascular and respiratory diseases	Submaximal exercise on the cycle ergometer	CO, NO_2_, O_3_, SO_2_, and PM_10_	Closed	Urban	Older adult volunteers without clinically cardiopulmonary disease were more vulnerable to factors related to autonomic control of the heart, inflammatory mediators, and circulating blood clotting factors when exposed to the fine effects of PM immediately after performing intermittent exercise than people of a similar age with COPD.
Pekkanen et al. (2002) [70]	Netherlands/Amsterdam, Germany/Erfurt, and Finland/Helsinki	Positive	Non-smoking older adults diagnosed with stable CAD	68.2 ± 6.5	♀ ♂	Cardiovascular diseases	Submaximal exercise test on a cycle ergometer	PM and NO_2_	Open	Urban	An association was observed between air pollution by fine and ultrafine particles and the risk of exercise-induced ST-segment depression in older people with CAD.
Gold et al. (2000) [71]	USA/Boston	Positive	Older volunteers	73.3 ± NR	♀ ♂	Cardiovascular diseases	Outdoor walk	PM_2.5_, PM_10_, O_3_, CO, NO_2_, and SO_2_	Open	Urban	Exposure to particulate matter and ozone can decrease vagal tone, resulting in reduced heart rate variability, both before and during a PA protocol.
Drechsler-Parks (1995) [37]	USA/Santa Barbara	Positive	Healthy non-smoking volunteers	65.9 ± 9	♀ ♂	Cardiovascular diseases	Exercise on a cycle ergometer or treadmill	O_3_ and NO_2_	Closed	Urban	Healthy seniors exposed to a mixture of 0.60 ppm NO_2_ and 0.45 ppm O_3_ had a significantly lower exercise-induced increase in cardiac output compared to when the same protocol was performed with exposure to filtered air or O_3_ alone.
Allred et al. (1989) [72]	USA/Baltimore, Downey, and St. Louis	Positive	Men with CAD	62 ± 1	♂	Cardiovascular diseases	Incremental treadmill test	CO	Closed	NR	An effect of CO on myocardial ischemia was objectively demonstrated by electrocardiographic changes during exercise. CO exposures produced carboxyhemoglobin levels of 2% and 3.9%, respectively. Low levels of carboxyhemoglobin exacerbated myocardial ischemia during graded exercise in individuals with CAD.
Reisenauer et al. (1988) [73]	USA/Seattle	Positive	Older volunteers	64.6 ± 6.36	♀ ♂	Respiratory diseases	Incremental intermittent exercise on the treadmill	O_3_	Closed	Urban	There was an increased risk of changes in lung function at close to ambient levels after exposure to O_3_ during exercise in healthy older adult subjects.
Drechsler-Parks et al. (1987) [74]	USA/Santa Barbara	Positive	Healthy non-smokers	62.85 ± 6.2	♀ ♂	Respiratory diseases	Cycle ergometer	O_3_	Closed	NR	O_3_ exposure induced significant decreases in forced vital capacity and forced expiratory volume at 1 and 3 s post-exposure compared with pre-exposure during exercise to induce minute ventilation.

♀: female; ♂: male; PA: physical activity; CAD: coronary artery disease; COPD: chronic obstructive pulmonary disease; SD: standard deviation; USA: USA of America; Min.: minimum; Max.: maximum; NO_x_: nitrogen oxides; NO_2_: nitrogen dioxide; NR: not reported; $\bar{x}$: average; PM: particulate matter; CO: carbon monoxide; O_3_: ozone; SO_2_: sulfur dioxide; ppm: part per million. * There was no mean and standard deviation.

**Table 6:** Sample characteristics (sex and age), pollutants, physical activity, health outcomes, and ambiguous findings about pollutants in the health of older adults during PA.

Authors, Year	Country City	Positive, Negative, and Ambiguous	Population	Age $(\bar{x\;}$ ± SD/Min-Max)	Sex	Health Outcomes	PA Type	Exposure (Pollutants)	Environment-Open/Closed	Environment by location (Urban/Rural/Commercial)	Main Findings
Ao et al. (2022) [75]	China/Sichuan, Chongqing, Guizhou, Yunnan	Ambiguous	Seniors from five provinces in southwest China	60.54 ± 7.50	♀ ♂	Chronic diseases	General AF	PM_10_, PM_2.5_, PM_1.0_	Open	NR	For the prevention of type 2 diabetes in the older adults, the health benefits of PA may outweigh the harm caused by air pollution, except in extreme air pollution situations, and that PA levels should not exceed 40 MET- h/d when the air quality of residence is severe.
Stieb et al. (2021) [76]	Canada/Ontario	Ambiguous	Healthy older adults	NR/55–81	♀ ♂	Cardiovascular and respiratory diseases and overall health outcomes	Daily light PA (e.g., walking)	CO, NO_2_, O_3_, PM_2.5_, SO_2_	Open	NR	Associations between air pollution and weekly pre-exercise heart rate and measures of heart rate variability, as well as urinary malonaldehyde, were consistently stronger in the group that performed outdoor PA (1.4% increase in heart rate). However, these differences cannot plausibly be attributed to the intervention, as there were only two days of intervention.
Besser et al. (2021) [77]	USA/Forsyth County (Forsyth County), North Carolina; New York, Baltimore, Maryland Saint Paul, Minnesota Chicago, Illinois, Los Angeles, California	Ambiguous	American seniors without dementia	67 ± 8	♀ ♂	Mental disorders	Reported moderate/vigorous activities achieved by any means (e.g., purposeful exercise, housework, lawn/garden, day care, transportation, work)	PM_2.5_	Open	Urban	Depressive symptoms, total PA per week and exposure to PM_2.5_ did not mediate the association between proportion of park space and global cognition.
Huang et al. (2021) [38]	China	Ambiguous	Adults aged 50 and over in the first wave of the Global Aging and Adult Health Study in China during 2007–2010	50/NR	♀ ♂	Cardiovascular diseases	General PA	PM_2.5_	Open	Urban/rural	We found a direct and negative association of residential green with hypertension prevalence for rural participants, but not for urban participants. The association of green with hypertension was completely mediated by PM_2.5_ concentrations (without any mediating effect of physical activity and BMI) in urban areas. In contrast, the association was mediated by concentrations of PM_2.5_, PA and others not observed in rural areas.
Zhou et al. (2020) [78]	China/Guangzhou	Ambiguous	Who lived in Guangzhou for more than 6 months	60/NR	♀ ♂	Overall health outcomes/mental disorders	General PA	PM_2.5_	Open	External (urban/rural/commercial)	Neighborhood green has a positive association with PA in the older adults, which is positively linked to their physical health. There was no significant relationship between PA and the investigated PM_2.5._
Roe et al. (2020) [79]	USA/Richmond	Ambiguous	Older adults/American seniors	64.8 ± NR	♀ ♂	Mental disorders	Walk on two routes: urban green and urban gray	PM_2.5_	Open	Urban	Walking on a green route (lower degree of pollution) provided more benefits for the well-being of the older adults, while walking on a gray area (higher degree of pollution) increased a physiological factor related to stress.
Nuyts et al. (2019) [80]	Belgium/Louvain	Ambiguous	Healthy older adults	58–76 *	♀ ♂	Mental disorders	General PA	NO_2_	Open	Urban	Pollution demonstrated a negative effect on positive affect when average daily steps were low. Moderate PA attenuated the effect of NO_2_ on positive affect. However, short-term exposure to NO_2_, considered the products of combustion related to traffic, can produce non-pathological changes in mood in healthy older people.
Balmes et al. (2019) [81]	USA/Rochester, Chapel Hill, and San Francisco	Ambiguous	Older non-smoking adults	59.9 ± 4.5	♀ ♂	Other diseases	Moderate intermittent physical exercise	O_3_	Closed	Urban	Exposure to low-level O_3_ during exercise in healthy older adults had no effects on systemic inflammation, oxidative stress, or prothrombotic status.
Rich et al. (2018) [82]	USA/Rochester, Chapel Hill, and San Francisco	Ambiguous	Healthy seniors and non-smokers	59.9 ± 4.5	♀ ♂	Cardiovascular diseases	Moderate intermittent physical exercise	O_3_	Closed	Urban	There was no convincing evidence for acute effects at random 3-hour exposures to 0.70 and 120 ppb O_3_ alternating 15 min of moderate exercise with 15 min of rest on cardiovascular function.
Sinharay et al. (2018) [83]	England/London	Ambiguous	Older people with ischemic heart disease or COPD and healthy volunteers	65.4 ± 1.2	♀ ♂	Respiratory diseases	Walk	PM_2.5_, PM_10_, carbon black, NO_2_, and ultrafine particles	Open	Urban	Patients with COPD reported more coughing, sputum, shortness of breath, and wheezing after walking on the most polluted road compared to the polluted one. In all groups of participants, walking in *Hyde Park* generated several beneficial responses to the cardiopulmonary system; such effects were mitigated after the walk on *Oxford Street*.
Arjomandi et al. (2018) [84]	USA/Rochester, Chapel Hill, and San Francisco	Ambiguous	Healthy, non-smoking older adults	59.9 ± 4.5	♀ ♂	Respiratory diseases	Walk	O_3_	Closed	Urban	Exposure to O_3_ attenuated the effects of physical exercise on lung function and minute ventilation. O_3_ exposure caused a marginally significant increase in airway inflammatory markers in a concentration-dependent manner. Plasma levels of a marker of airway epithelial injury also increased with O_3_ concentration.
Dewulf et al. (2016) [85]	Belgium/Ghent	Ambiguous	Older adult residents of Ghent	61.7 ± NR	♀ ♂	General health results	AF in transport-walking and cycling	NO_2_	Open	Urban	The high ventilation rate of cycling resulted in the highest inhaled dose of NO_2_, when compared to other modes of transport such as walking, driving and stationary. However, the daily proportion of cycling activity was relatively low and the influence of this activity on the total amount of inhaled NO_2_ is small.
Bartell et al. (2013) [86]	USA/Los Angeles	Ambiguous	Non-smoking older adults diagnosed with CAD	83.3 ± 5.9	♀ ♂	Cardiovascular diseases	General PA	PM_2.5_, black carbon, O_3_, CO, NO_x_, elemental carbon, organic carbon	Open	Urban	The data support the hypothesis that particle exposures may increase the risk of ventricular tachycardia in older adults patients with CAD. Heart rate variability was not associated with exposure in most of our participants. Evidence of the effects of pollutants on PA measures was limited and inconclusive.
Babb (1997) [36]	USA/Dallas	Ambiguous	Older people with normal lung function	68 ± 2	♀ ♂	Respiratory diseases	Incremental test on cycle ergometer	CO_2_e and He-O_2_	Closed	NR	Mechanical ventilatory limitations, even minimal, can attenuate the ventilatory response to intense exercise as much as possible when subjects were breathing room air or when breathing 3% CO_2,_ despite the large increase in chemical impulse for minute ventilation. However, the attenuation of ventilation per minute is not sufficient to limit exercise capacity, as all subjects showed normal exercise capacity when breathing room air and breathing 3% CO_2_.
Drechsler-Parks (1995) [87]	USA/Santa Barbara	Ambiguous	Healthy non-smoking volunteers	65.3 ± 4.12	♂	Respiratory diseases	Treadmill exercise	O_3_	Closed	NR	There were no significant differences between the responses to the three O_3_ exposures during walking on a motorized treadmill. All three O_3_ exposures induced small, statistically significant decreases in forced vital capacity and forced expiratory volume, but no significant changes in forced expiratory flow rate between 25 and 75%. The data suggest possible saturation of the pulmonary response mechanism with a low degree of functional impairment in older men.
Bedi et al. (1989) [88]	USA/Santa Barbara	Ambiguous	Healthy non-smoking volunteers	67.9 ± 6.6	♀ ♂	Respiratory diseases	Exercise on a cycle ergometer or treadmill	O_3_	Closed	NR	There were no significant changes in any symptom question, despite a three-fold increase in the total number of symptoms reported during O_3_ exposure. An adaptive response was observed on the third day of exposure that persisted through the 72-hour period of non-exposure based on the absence of significant differences between those days and the day of PA.
Drechsler-Parks et al. (1987) [89]	USA/Santa Barbara	Ambiguous	Healthy non-smokers	62.8 ± 6.2	♀ ♂	Respiratory diseases	Cycle ergometer	NO_2_	Closed	NR	There were no statistically significant differences between the responses of men and women aged 55 years and older to exposure to filtered air or NO_2_, in forced vital capacity and heart rate during each exercise period.
Rondinelli et al. (1987) [33]	USA/Washington	Ambiguous	Healthy male volunteers	64/55–73	♂	Respiratory diseases	Treadmill walk	SO_2_	Closed	NR	The present study does not support the hypothesis that older adults show greater bronchial reactivity to inhaled SO_2_ during moderate exercise on a treadmill than normal adolescents, although the former showed a slightly greater response. This effect was short-lived and of small magnitude. Clearly, these changes do not represent a life-threatening pulmonary response to SO_2._

♀: female; ♂: male; PA: physical activity; CAD: coronary artery disease; CO: carbon monoxide; CO_2_ e: carbon dioxide equivalent; COPD: chronic obstructive pulmonary disease; SD: standard deviation; USA: United States of America; He-O_2_: helium-oxygen; BMI: body mass index; Min.: minimum; Max: maximum; PM: particulate matter; NO_x_: nitrogen oxides; NO_2_: nitrogen dioxide; NR: not reported; BP: blood pressure; $\bar{x}$: average; O_3_: ozone; SO_2_: sulfur dioxide; ppb: part per billion. * There was no mean and standard deviation.

**Table 7:** MMAT quality appraisal results *.

Authors, Year	2. Randomized Controlled Trials					3. Non-Randomized Studies				
	2.1. Was Randomization Appropriately Performed?	2.2. Were the Groups Comparable at Baseline?	2.3. Were There Complete Outcome Data?	2.4. Were Outcome Assessors Blinded to the Intervention Provided?	2.5 Did the Participants Adhere to the Assigned Intervention?	3.1. Were the Participants Representative of the Target Population?	3.2. Were Measurements Appropriate Regarding Both the Outcome and Intervention (or Exposure)?	3.3. Were There Complete Outcome Data?	3.4. Were the Confounders Accounted for in the Design and Analysis?	3.5. During the Study Period, Was the Intervention Administered (or Exposure Occurred) as Intended?
Ao et al. (2022) [75]						Yes	Yes	Yes	Yes	Yes
Liu et al. (2022) [43]						Yes	Can’t tell	Yes	Can’t tell	Yes
Yao et al. (2022) [44]						Yes	Can’t tell	Yes	Yes	Yes
Wu et al. (2022) [45]						Yes	Yes	Yes	Yes	Yes
Yu et al. (2021) [15]						Yes	Yes	Yes	Yes	Yes
Bagheri et al. (2021) [39]						Yes	No	Yes	Yes	Yes
Stieb et al. (2021) [76]	Can’t tell	Yes	Yes	Can’t tell	Yes					
Besser et al. (2021) [77]						Yes	Can’t tell	Yes	Can’t tell	Yes
Huang et al. (2021) [38]						Yes	Yes	Yes	Yes	Yes
Cassilhas et al. (2022) [41]	Yes	Yes	Can’t tell	Can’t tell	Yes					
Ran et al. (2021) [46]						Yes	Yes	Yes	Yes	Yes
Kim et al. (2020) [35]						Yes	Yes	Yes	Yes	Yes
Zhou et al. (2020) [78]						Yes	No	Can’t tell	Can’t tell	Can’t tell
Sun et al. (2020) [47]						Yes	Yes	Yes	Yes	Yes
Molina-Sotomayor et al. (2020) [48]						Yes	Yes	Yes	Can’t tell	Can’t tell
Elliott et al. (2020) [34]						Yes	Yes	Yes	Yes	Yes
Wang et al. (2020) [49]						Yes	Can’t tell	Yes	Yes	Can’t tell
Roe et al. (2020) [79]						Yes	Yes	Can’t tell	Can’t tell	Yes
Nuyts et al. (2019) [80]						Yes	Yes	Yes	Yes	Yes
Molina-Sotomayor et al. (2019) [50]						Yes	Yes	Yes	Can’t tell	Yes
Chen et al. (2019) [51]						Yes	Yes	Can’t tell	Yes	Can’t tell
Balmes et al. (2019) [81]						Yes	No	Can’t tell	Can’t tell	Can’t tell
Zwart et al. (2018) [57]						Yes	Yes	Can’t tell	Yes	Yes
Stieb et al. (2018) [58]						Yes	Yes	Can’t tell	Can’t tell	Yes
Rich et al. (2018) [82]	Can’t tell	Yes	Can’t tell	Can’t tell	Yes					
Kubesch et al. (2018) [52]						Yes	No	Can’t tell	Yes	Yes
Sinharay et al. (2018) [83]	Can’t tell	Yes	Can’t tell	Can’t tell	Yes					
Arjomandi et al. (2018) [84]	Can’t tell	Yes	Yes	Can’t tell	Yes					
Yu et al. (2017) [59]						Yes	Yes	Yes	No	Yes
Stieb et al. (2017) [60]						Yes	Yes	Can’t tell	Can’t tell	Yes
Endes et al. (2017) [53]						Can’t tell	Can’t tell	Yes	Yes	Yes
Dewulf et al. (2016) [85]						No	Yes	Can’t tell	No	Can’t tell
Salicio et al. (2016) [61]						Yes	Can’t tell	Can’t tell	No	Can’t tell
Andersen et al. (2015) [54]						Yes	Can’t tell	Yes	Yes	Can’t tell
Alahmari et al. (2015) [62]						Yes	Yes	Yes	Can’t tell	Yes
Kim et al. (2015) [55]						Yes	Can’t tell	Yes	Yes	Yes
Bartell et al. (2013) [86]						Yes	Yes	Yes	Yes	Yes
Delfino et al. (2011) [63]						Yes	Yes	Yes	Yes	Yes
Delfino et al. (2010) [64]						Yes	Yes	Yes	Yes	Yes
Lanki et al. (2008) [65]						Yes	Yes	Yes	Yes	Yes
Wong et al. (2007) [56]						Yes	Can’t tell	Yes	Yes	Yes
Lanki et al. (2006) [66]						Yes	Yes	Yes	Yes	Yes
Gold et al. (2005) [67]						Yes	Yes	Can’t tell	Yes	Yes
Gong et al. (2005) [42]						Can’t tell	Yes	Can’t tell	Can’t tell	Yes
DeMeo et al. (2004) [68]						Yes	Yes	No	Can’t tell	Can’t tell
Gong et al. (2004) [69]						Can’t tell	Yes	Can’t tell	Can’t tell	Yes
Babb et al. (2003) [40]						No	Yes	Can’t tell	Can’t tell	Can’t tell
Pekkanen et al. (2002) [70]						Yes	Yes	Yes	Can’t tell	Yes
Gold et al. (2000) [71]						Yes	Yes	Can’t tell	Yes	Yes
Babb (1997) [36]						No	Can’t tell	Can’t tell	Can’t tell	Can’t tell
Drechsler-Parks (1995) [37]						No	Yes	Can’t tell	Can’t tell	Can’t tell
Drechsler-Parks (1995) [87]						No	Yes	Can’t tell	Can’t tell	Can’t tell
Bedi et al. (1989) [88]						No	Yes	Can’t tell	Can’t tell	Can’t tell
Allred et al. (1989) [72]						No	Yes	Can’t tell	Can’t tell	Can’t tell
Reisenauer et al. (1988) [73]						Yes	Yes	No	Can’t tell	Can’t tell
Drechsler-Parks et al. (1987) [89]						No	Can’t tell	Can’t tell	Can’t tell	Can’t tell
Drechsler-Parks et al. (1987b) [74]						No	Can’t tell	Can’t tell	Can’t tell	Can’t tell
Rondinelli et al. (1987) [33]						Can’t tell	No	Can’t tell	Can’t tell	Can’t tell

* The Mixed Methods Appraisal Tool questions are presented in the form of statements in Table 7.

**Table 8:** Recommendations for policymakers, health professionals, researchers, and practitioners of physical activities.

Politicians, Health Professionals, and Researchers	Practitioners of Physical Activities
More research on the combined effects of PMs and PA on diabetes is urgently needed.	It is suggested that aerobic exercise in the open air be avoided when there are high levels of PM, so as not to affect the positive effects of the practice, in the levels of neuro factors derived from the brain.
We suggest that urban planners design green spaces in neighborhoods where older adults can nurture and communicate with each other. They can also plant trees along sidewalks to provide a desirable walking environment for older adults and recreational infrastructure under shaded trees to increase community comfort among older adults. Finally, green spaces can be developed in communities with a high proportion of senior citizens, especially for those belonging to low-income groups.	It is recommended that physical activities be carried out in places with green spaces, as this encourages the practice, mitigates air pollution, increases social cohesion in localities/neighborhoods, and improves satisfaction with life;
Studies with a window of exposure to PM_2.5_ greater than or equal to 180 days are recommended on the effects on depressive symptoms.	Moderate-to-vigorous physical activity (MVPA) is recommended for older adults wherever they live, although practicing MVPA in an environment that considers air pollutant reduction is important to maximize the health benefits of MVPA in diabetes.
Experimental studies are needed to clarify the specific mechanisms among older people with low levels of physical activity and vitamin D deficiency, exposed to high levels of PM_2.5_, presenting high chances of depression.	For countries or regions with high pollution, especially those cities with poor sunlight or a high prevalence of vitamin D deficiency, additional vitamin D supplementation and physical activity are recommended. This can help to mitigate the adverse effects of exposure to PM_2.5_ on health conditions and may hinder the occurrence of major depressive disorder.
Policies and strategies are needed to reduce O_3_ exposure to ensure that the health benefits of physical activity are not diminished by higher O_3_ levels in susceptible populations, such as older Hispanics and type 2 diabetics.	The use of active transport is recommended to reduce sedentary levels, such as bicycles and other modes; however, it is recommended only when the concentration of air pollution is reduced in order to limit negative impacts on health.
Structural change policies to reduce levels of exposure to pollutants in built and social environments, such as parks with walking paths or recreational and sporting infrastructure environments, are potential opportunities to delay or reduce the risk of dementia.	Physical activity is recommended for people with dementia or Alzheimer’s disease who live in relatively polluted areas.
Longitudinal studies are needed to provide a cause-and-effect association between hypertension and the practice of physical activity in green spaces in urban areas, mediated by air pollution, which can help policymakers and professionals to conduct effective interventions to prevent and control the prevalence of hypertension and the burden of concomitant diseases.	Avoiding physical activity when exposed to air pollution at chronic levels, as it results in mood swings and increases the risk of depression or anxiety disorders. However, it is suggested that when levels are medium/low, walking more than 9000 steps per day be carried out, mitigating the harmful effects of air pollution.
Future studies are needed to confirm the impacts of pollutants on indoor and outdoor exercise.	Older people, when performing physical exercises in external environments, when possible, should be monitored by professionals with greater attention to climatic conditions and pollution, suggesting the use of a portable monoximeter as a monitoring instrument.
It is necessary that the development of outdoor physical activity guidelines and emergency response plans for heavy air pollution be discussed.	We recommend, especially for the most susceptible populations, that they avoid increasing the absorption of atmospheric pollutants through simple measures, such as avoiding active displacement or exercise along busy main roads, as well as opting for less polluted alternatives, such as secondary roads, parks with green areas etc.
Future studies are needed to validate whether the positive health effects of moderate-to-vigorous physical activity (MVPA) outweigh the potential harmful effects due to increased exposure to air pollution during MVPA.	It is suggested that healthy people, as well as those with chronic cardiorespiratory disorders, should minimize walking on streets with high levels of pollution, as this reduces or even reverses the cardiorespiratory benefits of exercise. Instead, walking exercise should be enjoyed in urban green areas away from high-density traffic.
More research is needed to expand knowledge and understanding of the protective effects that planned physical activity have on cognitive function and cardiovascular adaptations in the presence of high concentrations of particulate matter in the atmosphere.	It is suggested that older people living in rural areas may benefit from reducing outdoor activity when air pollution levels are particularly high, in order to reduce acute adverse cardiorespiratory effects.
Additional research is recommended with the aim of identifying points on the Air Quality Health Index (AQHI) scale to provide protective advice that optimizes the balance between reducing outdoor activity when needed, in order to reduce short-term health risks of air pollution, preserving the long-term benefits of outdoor physical activity.	
Current environmental levels of air pollution along busy streets are unacceptable and need to be controlled. It is important to impose policies and measures that can reduce traffic pollution so that each individual can enjoy the benefits of physical activity for health.	
Policy interventions are needed to reduce the level of air pollution in China’s urban areas.	
New studies with longitudinal designs and conducted in broader areas of exposure to PM_2.5_ are needed to verify the relationship between physical activity and body composition, determining the effects on muscle function.	
Additional research in large prospective cohorts is needed to assess whether the observed effect modification (moderate-to-vigorous physical activity confers a protective effect against the adverse vascular effects of air pollution in low-pollution environments) translates to high-pollution environments in megacities in low- and middle-income countries.	
New studies that assess health impacts through epidemiological studies need to incorporate individual travel behavior and physical activity to measure the inhaled dose of air pollution. This can be performed accurately using GPS and accelerometer data.	
Training physical education professionals to care for the practice of exercises with older adults in polluted environments can minimize the health risks of this population.	

## Data Availability

No new data were created or analyzed in this review. Data sharing does not apply to this article.

## Supplementary Materials

The following supporting information can be downloaded at https://www.mdpi.com/article/10.3390/ijerph20043506/s1. Table S1: Template for a Mapping Study Protocol. Table S2: Literature search strategy on PubMed. Table S3: Titles of included reviews (58).

## Abbreviations

AQHI	air quality health index
BMI	body mass index
CAD	coronary artery disease
CO	carbon monoxide
CO_2_e	carbon dioxide equivalent
COPD	chronic obstructive pulmonary disease
DV	standard deviation
GPS	global positioning system
H	hours
He	helium
He-O_2_	helium–oxygen
Max	maximum
MET	metabolic equivalent of task
Min.	minimum
MMAT	mixed methods appraisal tool
MVPA	moderate to vigorous physical activity
NO₂	nitrogen dioxide
Nox	nitrogen oxides
NR	not reported
O_3_	ozone
PA	physical activity
Pas	physical activities
PECOS	population, exposure, comparator, outcome, study design
PM	particulate matter
PPB	part per billion
PPM	part per million
RQs	research questions
SO₂	sulfur dioxide
TSP	total suspended particles
UFP	ultrafine particles
USA	United States of America
VOPs	volatile organic pollutants
WHO	World Health Organization
WK	week
