# Supplementary material for: Effects of Air Pollution on the Health of Older Adults during Physical Activities: Mapping Review
Source: Int J Environ Res Public Health. 2023 Feb 16;20(4):3506. doi: 10.3390/ijerph20043506 (PMC9960154; doi:10.3390/ijerph20043506)
Supplement: Supplementary file 1 [file ijerph-20-03506-s001.zip › ijerph-2203696-supplementary.pdf]

Supplementary Material

**Supplementary Material 1**	Table S1. Template for a Mapping Study Protocol	page 2-3
**Supplementary Material 2**	Table S2. Literature search strategy on PubMed	page 4
**Supplementary Material 3**	Table S3. Titles of included reviews (58)	page 5-6

Table S1. Template for a Mapping Study Protocol

Section	Recommendation	Line #
**1**	**Change Record**<br>This should be a list or table summarizing the main updates and changes embodied in each version of the protocol and (where appropriate), the reasons for these.	NA
**2**	**Background**<br>a) explain why there is a need for a study on this topic<br>b) identify the topic that is to be 'scoped' in the study<br>c) specify any research questions that will be addressed<br>d) if extending previous research on the topic, explain why a new study is needed	a) 47-97<br>b) 83-97<br>c) 57-82<br>d) 66-97
**3**	**Search Strategy**<br>a) specify and justify basic strategy: manual search, automated search, or mixed<br>b) for automated searches, specify search terms and compounds of these (and record results of any prototyping ofthe search strings)<br>c) for automated searches, identify resources to be used (digital libraries and search engines)<br>d) for manual searches, identify the journals and conferences to be searched<br>e) specify the time period to be covered by the review and any reasons for your choice<br>f) identify any ancillary search procedures, e.g. asking leading researchers or research groups, or accessing their web sites; or checking reference lists of primary studies<br>g) specify how the search process is to be evaluated (e.g. against a known subset of papers; or against the results from a previous systematic review or mapping study)	a) 128-139<br>b) 133 (Table 1)<br>c) 128-129<br>d) N/A<br>e) 130<br>f) N/A<br>g) 118-120
**4**	**Selection Criteria**<br>**a)** identify the *inclusion* criteria for primary studies<br>**b)** identify the *exclusion* criteria<br>**c)** define how selection will be undertaken (roles of analysts)<br>**d)** define how agreement among analysts will be evaluated<br>**e)** define how any differences between analysts will be resolved	a) 155 (table 2)<br>b) 155 (table 2)<br>c) 142-143<br>d) 142-149<br>e) 142-143
**5**	**Data Extraction**<br>**a)** design data extraction form (and check via a dry run)<br>**b)** specify the strategy for extracting the data and the form (paper, on-line etc.)<br>**c)** identify how the data extraction process is to be undertaken and validated, particularly any data that require numerical calculations, or are subjective	a) 159-164<br>b) 159-187<br>c) 159-164
**6**	**Synthesis**<br>**a)** specify the categorization schemes to be used<br>**b)** assess the threats to validity (construct, internal, external), particularly constraints on the search process and deviations from standard practice	a) 165-187<br>b) N/A
**7**	**Study Limitations**<br>Specify residual validity issues including potential conflicts of interest (i.e. that are inherent in the context of the study, rather than arising from the plan).	519
**8**	**Reporting**	559 (Table 8)

	Identify target audience, relationship to other studies, planned publications, authors of the publications.	
**9**	**Schedule**<br>Provide time estimates for all of the major steps.	N/A

N/A: Not applied.

Supplementary material 2.

Table S2. Literature search strategy on PubMed

Estratégia de pesquisa no PubMed		
Busca	Query	Itens encontrados
#1	("aged"[MeSH Terms] OR "aged"[All Fields] OR "elderly"[All Fields] OR "elderlies"[All Fields] OR "elderly s"[All Fields] OR "elderlys"[All Fields] OR ("aged"[MeSH Terms] OR "aged"[All Fields]) OR ("aging"[MeSH Terms] OR "aging"[All Fields] OR "ageing"[All Fields]) OR "aged 80 and over"[All Fields] OR "older adults"[All Fields] OR "older women"[All Fields] OR "older men"[All Fields] OR ("aging"[MeSH Terms] OR "aging"[All Fields] OR "senescence"[All Fields] OR "senesce"[All Fields] OR "senesced"[All Fields] OR "senescences"[All Fields] OR "senescent"[All Fields] OR "senescents"[All Fields] OR "senesces"[All Fields] OR "senescing"[All Fields]) OR "Oldest Old"[All Fields] OR "Old Adults"[All Fields] OR "nonagenarian*"[All Fields] OR "octogenarian*"[All Fields] OR "centenarian*"[All Fields]) AND ("air pollution"[All Fields] OR "air pollutant*"[All Fields] OR "air quality"[All Fields] OR "particulate matter"[All Fields] OR "PM10"[All Fields] OR "PM2.5"[All Fields] OR "carbon monoxide"[All Fields] OR "carbon dioxide"[All Fields] OR "ozone"[All Fields] OR "nitrogen dioxide"[All Fields] OR "sulfur dioxide"[All Fields] OR "traffic-related air pollution"[All Fields]) AND ("exercise"[All Fields] OR "physical exercise*"[All Fields] OR "physical activity"[All Fields] OR "physical exertion"[All Fields] OR "physical training"[All Fields] OR "sport*"[All Fields] OR "resistance training"[All Fields] OR "strength training"[All Fields] OR "aerobic exercise"[All Fields] OR "aerobic fitness"[All Fields] OR "athlete"[All Fields] OR "athletic performance"[All Fields] OR "running"[All Fields] OR "cycling"[All Fields])	3585

Supplementary material 3.

Table S3. Titles of included reviews

1.	The joint effects of physical activity and air pollution on type 2 diabetes in older adults
2.	Can multiple pathways link urban residential greenspace to subjective well-being among middle-aged and older Chinese adults?
3.	Association of depressive symptoms with ambient PM(2.5) in middle-aged and elderly Chinese adults: A cross-sectional study from the China health and Retirement Longitudinal Study wave 4
4.	Fine particulate matter, vitamin D, physical activity, and major depressive disorder in elderly adults: Results from UK Biobank
5.	Ozone Exposure, Outdoor Physical Activity, and Incident Type 2 Diabetes in the SALSA Cohort of Older Mexican Americans
6.	The Impact of Built and Social Environmental Characteristics on Diagnosed and Estimated Future Risk of Dementia
7.	Associations between air pollution and cardio-respiratory physiological measures in older adults exercising outdoors
8.	Associations Between Neighborhood Park Access and Longitudinal Change in Cognition in Older Adults: The Multi-Ethnic Study of Atherosclerosis
9.	Greenness-air pollution-physical activity-hypertension association among middle-aged and older adults: Evidence from urban and rural China
10.	Indoor aerobic exercise reduces exposure to pollution, improves cognitive function, and enhances BDNF levels in the elderly
11.	The joint association of physical activity and fine particulate matter exposure with incident dementia in elderly Hong Kong residents
12.	Association of combined effects of physical activity and air pollution with diabetes in older adults
13.	Association Pathways Between Neighborhood Greenspaces and the Physical and Mental Health of Older Adults-A Cross-Sectional Study in Guangzhou, China
14.	Benefits of physical activity not affected by air pollution: a prospective cohort study
15.	Effects of Progressive Resistance Training on Cognition and IGF-1 Levels in Elder Women Who Live in Areas with High Air Pollution
16.	Interaction between long-term exposure to fine particulate matter and physical activity, and risk of cardiovascular disease and overall mortality in U.S. women
17.	The longitudinal relationship between exposure to air pollution and depression in older adults
18.	The Urban Built Environment, Walking and Mental Health Outcomes Among Older Adults: A Pilot Study
19.	Air pollution and self-perceived stress and mood: A one-year panel study of healthy elderly persons
20.	Effects of Cardiorespiratory Exercise on Cognition in Older Women Exposed to Air Pollution
21.	Effects of PM2.5 on Skeletal Muscle Mass and Body Fat Mass of the Elderly in Taipei, Taiwan
22.	Ozone effects on blood biomarkers of systemic inflammation, oxidative stress, endothelial function, and thrombosis: The Multicenter Ozone Study in oldEr Subjects (MOSES)
23.	Air Pollution and Performance-Based Physical Functioning in Dutch Older Adults
24.	Cardiorespiratory Effects of Air Pollution in a Panel Study of Winter Outdoor Physical Activity in Older Adults
25.	Cardiovascular function and ozone exposure: The Multicenter Ozone Study in oldEr Subjects (MOSES)
26.	Effects of Leisure-Time and Transport-Related Physical Activities on the Risk of Incident and Recurrent Myocardial Infarction and Interaction With Traffic-Related Air Pollution: A Cohort Study
27.	Respiratory and cardiovascular responses to walking down a traffic-polluted road compared with walking in a traffic-free area in participants aged 60 years and older with chronic lung or heart disease and age-matched healthy controls: a randomised, crossover study
28.	Respiratory Responses to Ozone Exposure MOSES (The Multicenter Ozone Study in Older Subjects)
29.	Ambient Fine Particulate Matter Air Pollution and Physical Activity: A Longitudinal Study of University Retirees in Beijing, China
30.	Cardio-Respiratory Effects of Air Pollution in a Panel Study of Outdoor Physical Activity and Health in Rural Older Adults
31.	Is physical activity a modifier of the association between air pollution and arterial stiffness in older adults: The SAPALDIA cohort study
32.	Dynamic assessment of inhaled air pollution using GPS and accelerometer data
33.	Environmental variables and levels of exhaled carbon monoxide and carboxyhemoglobin in elderly people taking exercise

34.	A Study of the Combined Effects of Physical Activity and Air Pollution on Mortality in Elderly Urban Residents: The Danish Diet, Cancer, and Health Cohort
35.	Influence of weather and atmospheric pollution on physical activity in patients with COPD
36.	Physical Activity- and Alcohol-dependent Association Between Air Pollution Exposure and Elevated Liver Enzyme Levels: An Elderly Panel Study
37.	Particulate Air Pollution, Ambulatory Heart Rate Variability, and Cardiac Arrhythmia in Retirement Community Residents with Coronary Artery Disease
38.	Electrocardiographic ST-Segment Depression and Exposure to Traffic-Related Aerosols in Elderly Subjects with Coronary Artery Disease
39.	Traffic-related Air Pollution and Blood Pressure in Elderly Subjects With Coronary Artery Disease
40.	Hourly variation in fine particle exposure is associated with transiently increased risk of ST segment depression
41.	Does regular exercise protect against air pollution-associated mortality?
42.	Can we identify sources of fine particles responsible for exercise-induced ischemia on days with elevated air pollution? The ULTRA study
43.	Air pollution and ST-segment depression in elderly subjects
44.	Respiratory responses to exposures with fine particulates and nitrogen dioxide in the elderly with and without COPD
45.	Ambient air pollution and oxygen saturation
46.	Exposures of elderly volunteers with and without chronic obstructive pulmonary disease (COPD) to concentrated ambient fine particulate pollution
47.	Ventilatory response to exercise in aged runners breathing He-O2 or inspired CO2
48.	Particulate air pollution and risk of ST-segment depression during repeated submaximal exercise tests among subjects with coronary heart disease: the Exposure and Risk Assessment for Fine and Ultrafine Particles in Ambient Air (ULTRA) study
49.	Ambient pollution and heart rate variability
50.	Ventilatory response to exercise in subjects breathing CO2 or HeO2
51.	CARDIAC-OUTPUT EFFECTS OF O-3 AND NO2 EXPOSURE IN HEALTHY OLDER ADULTS
52.	THE DOSE-RESPONSE RELATIONSHIP IN OLDER MEN EXPOSED TO OZONE
53.	Adaptation by older individuals repeatedly exposed to 0.45 parts per million ozone for two hours
54.	Short-term effects of carbon monoxide exposure on the exercise performance of subjects with coronary artery disease
55.	Pulmonary response to ozone exposures in healthy individuals aged 55 years or greater
56.	Pulmonary function responses of older men and women to NO2
57.	Pulmonary function responses of older men and women to ozone exposure
58.	The effects of sulfur dioxide on pulmonary function in healthy nonsmoking male subjects aged 55 years and older
